# A preoperative predictive model based on multi-modal features to predict pathological complete response after neoadjuvant chemoimmunotherapy in esophageal cancer patients

**DOI:** 10.3389/fimmu.2025.1530279

**Published:** 2025-01-27

**Authors:** Yana Qi, Yanran Hu, Chengting Lin, Ge Song, Liting Shi, Hui Zhu

**Affiliations:** ^1^ Department of Radiation Oncology, Shandong Cancer Hospital and Institute, Shandong First Medical University and Shandong Academy of Medical Sciences, Jinan, Shandong, China; ^2^ Department of Pathology, Shandong Cancer Hospital and Institute, Shandong First Medical University and Shandong Academy of Medical Sciences, Jinan, Shandong, China; ^3^ The Second School of Clinical Medicine, Zhejiang Chinese Medical University, Hangzhou, Zhejiang, China; ^4^ Department of Radiology, Zhejiang Cancer Hospital, Hangzhou Institute of Medicine (HIM), Chinese Academy of Sciences, Hangzhou, Zhejiang, China

**Keywords:** pathological complete response, radiomics, pathomics, support vector machine, esophageal cancer (EC)

## Abstract

**Background:**

This study aimed to develop a multi-modality model by incorporating pretreatment computed tomography (CT) radiomics and pathomics features along with clinical variables to predict pathologic complete response (pCR) to neoadjuvant chemoimmunotherapy in patients with locally advanced esophageal cancer (EC).

**Method:**

A total of 223 EC patients who underwent neoadjuvant chemoimmunotherapy followed by surgical intervention between August 2021 and December 2023 were included in this study. Radiomics features were extracted from contrast-enhanced CT images using PyrRadiomics, while pathomics features were derived from whole-slide images (WSIs) of pathological specimens using a fine-tuned deep learning model (ResNet-50). After feature selection, three single-modality prediction models and a combined multi-modality model integrating two radiomics features, 11 pathomics features, and two clinicopathological features were constructed using the support vector machine (SVM) algorithm. The performance of the models were evaluated using receiver operating characteristic (ROC) analysis, calibration plots, and decision curve analysis (DCA). Shapley values were also utilized to explain the prediction model.

**Results:**

The predictive capability of the multi-modality model in predicting pCR yielded an area under the curve (AUC) of 0.89 (95% confidence interval [CI], 0.75-1.00), outperforming the radiomics model (AUC 0.70 [95% CI 0.54-0.85]), pathomics model (AUC 0.77 [95% CI 0.53-1.00]), and clinical model (AUC 0.63 [95% CI 0.46-0.80]). Additionally, both the calibration plot and DCA curves support the clinical utility of the integrated multi-modality model.

**Conclusions:**

The combined multi-modality model we propose can better predict the pCR status of esophageal cancer and help inform clinical treatment decisions.

## Introduction

Esophageal cancer (EC) is one of the major types of cancer worldwide, with surgical therapy being the primary treatment modality. However, outcomes are often suboptimal due to early postoperative recurrence (1, 2). Large-scale randomized clinical trials have shown that neoadjuvant chemoimmunotherapy followed by surgery is more effective than surgery alone for locally advanced EC. Preliminary findings suggest that esophagectomy following neoadjuvant chemoimmunotherapy achieves satisfactory efficacy and manageable safety, with pathologic complete response (pCR) rates ranging from 16.7% to 50.0% (3–5). Patients achieving pCR exhibit a more favorable long-term prognosis compared to those without pCR and may benefit from wait-and-see strategies (6–8). Nevertheless, a subset of patients does not respond to neoadjuvant chemoimmunotherapy, potentially incurring high drug costs and immunotherapy-related adverse events (irAEs). Therefore, accurately predicting the response to neoadjuvant chemoimmunotherapy before surgery is of great clinical significance as it could identify patients who are likely to benefit and inform appropriate and personalized treatment plans.

Radiomics, a promising method that extracts high-throughput quantitative data from medical images, has been successfully applied in precision diagnosis, treatment response prediction, and prognosis assessment for various types of malignancies (9, 10). Several radiomics- based models have been proposed to predict pCR in EC patients undergoing neoadjuvant chemoradiotherapy (11, 12).However, limited research has explored the utilization of radiomics in assessing the response to neoadjuvant chemoimmunotherapy, and there is a need to improve the prediction accuracy of existing models (13). An optimal strategy that incorporates additional dimensions is essential for enhancing the performance of prediction models.

With the advent of digital pathology, quantitative analyses based on artificial intelligence have increased in the field of EC, demonstrating strong performance in early diagnosis, treatment response assessment, and survival prediction (14–16). Unlike radiomics features, digital pathology provides insights into molecular characteristics or genetic patterns, potentially complementing tumor heterogeneity and improving the predictive accuracy of current models. However, to our knowledge, there is a lack of academic research integrating pretreatment CT-derived radiomics features with pathomics features derived from whole-slide imaging (WSI) for predicting pCR in EC.

In this retrospective study, we aimed to develop and compare a multi-modality machine learning model by integrating pretreatment CT radiomics, pathomics features, and clinical variables to predict pCR in esophageal cancer prior to the initiation of therapy.

## Materials and methods

### Patients

**Figure 1:**
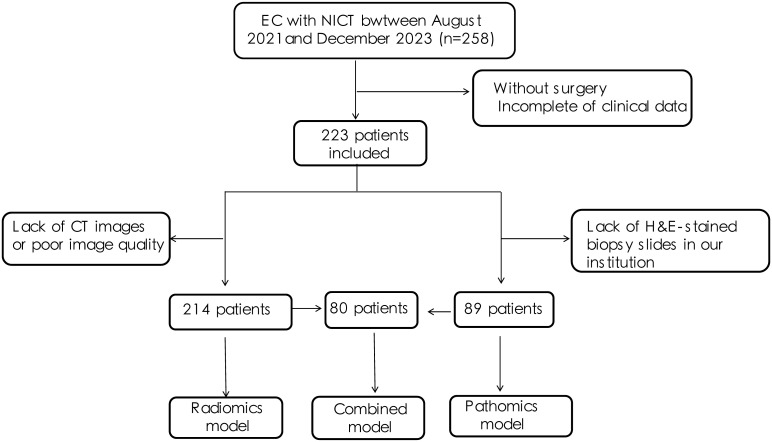
Flow diagram of patient cohort selection.

**Figure 2:**
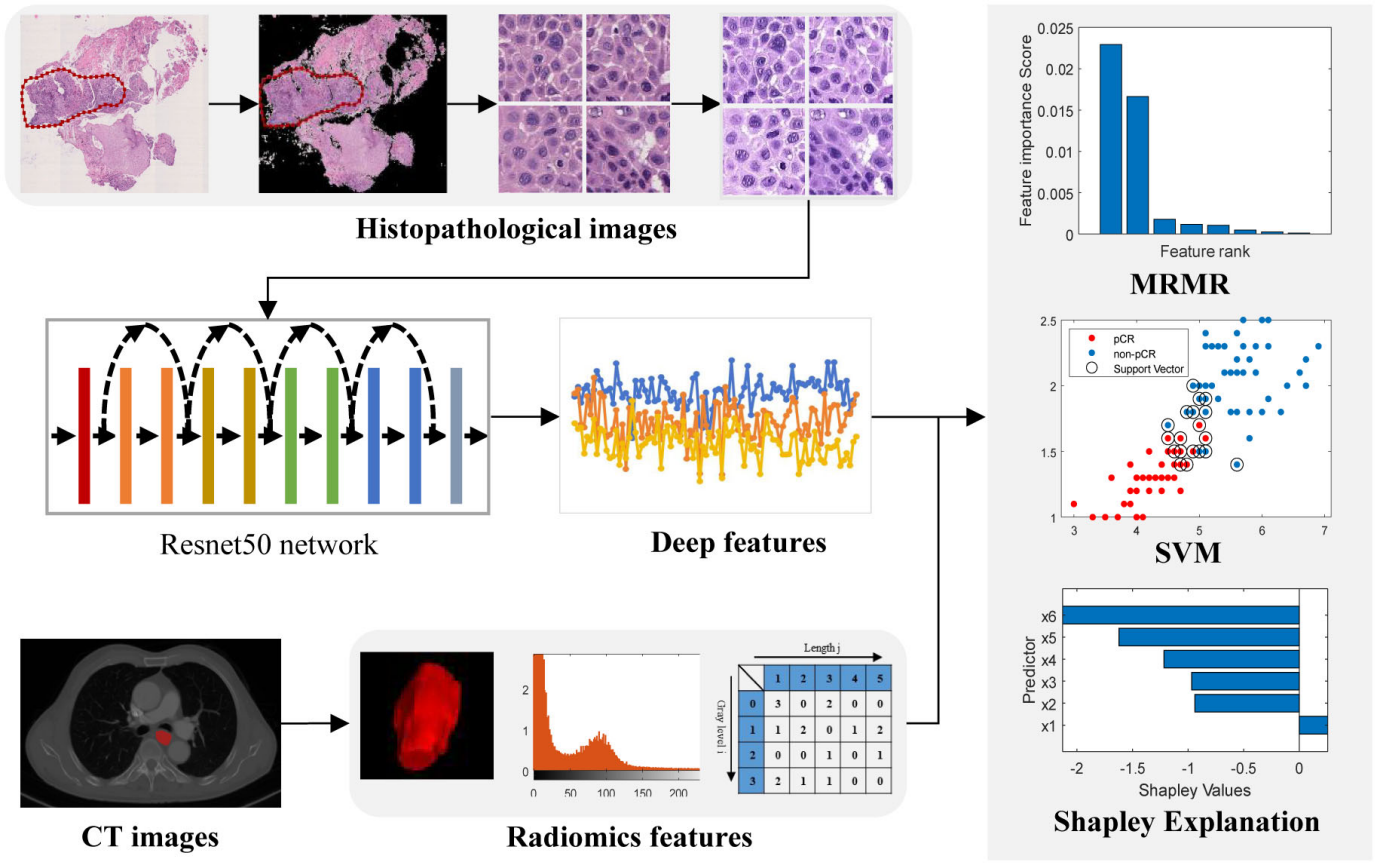
Workflow of the study. Images of pretreatment CT images and biopsy hematoxylin and eosin-stained slides were retrospectively retrieved and segmented for feature extraction. A two-sided sample t-test or U-test and the maximum relevance–minimum redundancy (MRMR) algorithm was used to select features. The top features with scores larger than zero were retained to develop a classifier model by a Support Vector Machine (SVM) method.

This retrospective study was approved by the Ethics Committee of Shandong Cancer Hospital and Institute and the requirement for written informed consent was waived. A specific flowchart of patient selection is displayed in **Figure 1**, and an overview of the entire architecture of the study is provided in **Figure 2**.

The inclusion criteria were as follows: (1) pathological biopsy confirming EC with no distant metastasis; (2) age between 18 and 75 years. The exclusion criteria were: (1) treatment with chemotherapy, radiotherapy, or other anticancer therapies prior to baseline CT scans; (2) synchronous tumors or a history of other malignancies; and (3) no surgical treatment following chemoimmunotherapy.

A total of 223 patients with histologically proven EC who underwent neoadjuvant chemoimmunotherapy and surgical treatment between August 2021 and December 2023 at Shandong Cancer Hospital and Institute were recruited for this study. Among these, 214 patients with pretreatment CT images were enrolled in the radiomics cohort to build a radiomics model. Additionally, 89 patients with WSI were used to construct the pathomics model. To further evaluate the ability of pathomics features to predict pCR when combined with CT-based radiomics and clinicopathological features, a combined multi-modality model was developed in 80 patients with both pretreatment CT and WSI. Patients were randomly allocated to the training and testing sets in a 3:1 ratio.

### Evaluation of tumor response

**Figure 3:**
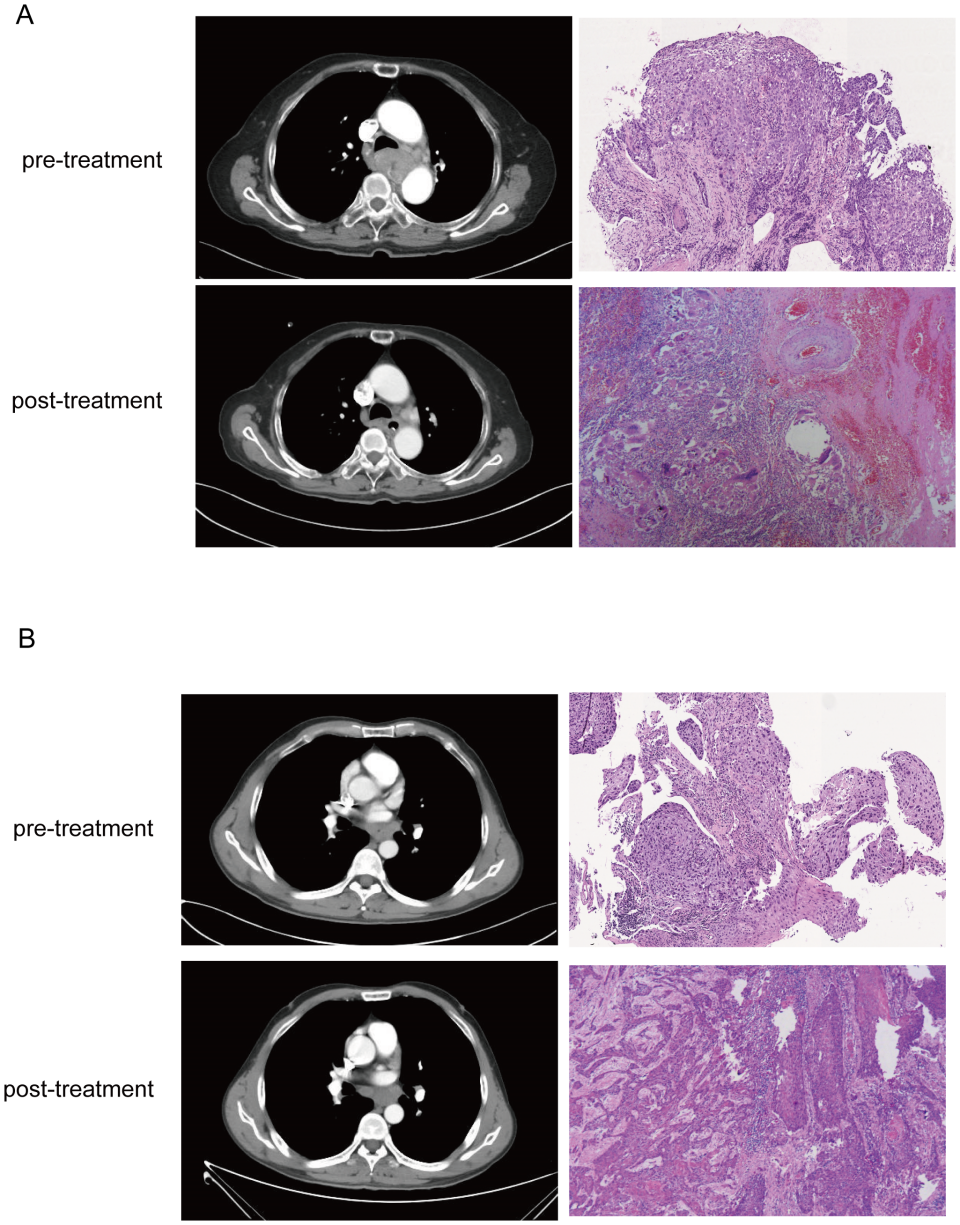
Corresponding CT images and WSIs from pCR **(A)** and non-pCR **(B)** before and after neoadjuvant treatment. In the CT image, it is seen that the tumor in the pCR have completely dissipated in the post-treatment image, and stromal tissue with no visible tumor cells was presented in the pathological images. But CT and histology images from non-pCR shows residual tumor cells but reduced compared to baseline.

The pathological response was assessed by examining the percentage of residual viable tumor cells in the resected specimens, based on the immune-mediated pathological response criteria (irPRC) (17, 18). Surgical specimens were evaluated by two pathologists who were blinded to clinical information and CT images (**Figure 3**). pCR was defined as the absence of viable tumor cells in both the primary tumor and the lymph nodes (**Figure 3A**).

### CT images and WSI acquisition

Contrast-enhanced CT scans were performed using a SIEMENS CT scanner before treatment. The scanning parameters were as follows: slice thickness of 5.0 mm, tube voltage of 120 kV, and tube current of 220 mA. An iodinated contrast agent (300 mg/mL) at a dose of 1.5 ml/kg of body mass was injected rapidly at a flow rate of 2 mL/s through the patient’s elbow vein using a high-pressure syringe. To maintain data consistency, only arterial phase CT images were collected. All acquired CT images were extracted from the institutional picture archiving and communication system (PACS) and saved in DICOM format.

H&E-stained slides from formalin-fixed paraffin-embedded biopsy tissues were used for pathological diagnosis. WSIs for analysis were scanned using a Panoramic SCAN II scanner at 40× objective magnification. All images were obtained in NDPI format for subsequent region of interest (ROI) annotation and pathological feature extraction.

### Image segmentation and feature extraction

Two experienced radiologists with 5 and 8 years of experience, who were blinded to the clinical and/or pathological data, manually delineated the regions of tumor on CT images with the ITK-SNAP software (version 3.6). The region of interest (ROI) was delineated to encompass the primary tumor volume in all slices. Before feature extraction, the CT image intensities were normalized to a scale of 2048 and re-binned by a width of 16 to avoid the influence of the sparse matrix on the calculation of texture features. And then the images of all patients were resampled to the same voxel size of 0.8 mm×0.8 mm×5 mm using the ‘sitkBSpline’ interpolator, in order to minimize the differences of images among patients. In total, 851 features, including shape, first-order, texture, and wavelet features, were computed and extracted using PyRadiomics (19) in Python 3.6.

For digital pathology image analysis, ASAP (version 2.1), an open-source software, was employed to annotate the tumor regions within the WSI. The original WSIs were converted from RGB to gray and a threshold of 220 was used to segment the tissue regions on the gray WSIs. Subsequently, the tissues in the tumor regions were subdivided into non-overlapping 224×224 tiles at a resolution of 0.5 μm/pixel (20× magnification). Tiles with less than 50% tissue were excluded from the analysis. Color normalization was performed on each tile to normalize the color variations caused by different staining and scanning of pathology images based on the Vahadane Method (20). The deep features of each tile were extracted using a pretrained ResNet50 backbone. The tiles were input into the ResNet50 backbone and the 2048 outputs at the fully connected layer were treated as 2048 deep features for each patch. For each deep feature, the median value of all tiles for one patient was treated as its feature value, resulting in 2048 deep features for each patient.

### Feature selection and model construction

Feature selection was performed separately for CT radiomics and pathomics features in the training set. The features were first normalized using z-scores and tested for normality. A two-sided sample t-test or U-test was applied to identify features showing significant differences between the pCR and non-pCR groups. Finally, features with p<0.1 were selected and further ranked using a maximum relevance-minimum redundancy (MRMR) algorithm (21, 22). Top features with scores greater than zero were retained for the classifier model. In addition to radiomics and pathomics features, critical clinical characteristics, including age, sex, smoking history, histology, treatment cycles, T-stage, N-stage, and clinical stage, were incorporated to select the feature set for constructing the multimodal prediction model.

Three single-modality prediction models—radiomics, pathomics, and clinical models—were constructed using a Support Vector Machine (SVM) approach. The SVM model, employing a radial basis function (RBF) kernel, was optimized through five-fold cross-validation using data from the training set. Subsequently, we developed and validated a multimodal model that integrated all selected radiomics features with pathomics features and significant clinical factors. The performance of these models in predicting pCR was assessed in both the training and testing sets using receiver operating characteristic (ROC) curve analysis and area under the curve (AUC). Youden’s index was used to determine the optimal threshold for converting model output scores into predicted classes. The accuracy, sensitivity, specificity, positive predictive value (PPV), and negative predictive value (NPV) were calculated. Calibration curves were used to evaluate the goodness-of-fit of the models, and decision curve analysis (DCA) was performed to quantify the net benefit to patients at different threshold probabilities, thus assessing the clinical utility of the predictive models developed in this study.

To further evaluate the contribution of the combined features to the multi-modality model prediction, the SHAP value was calculated to decompose the SVM model decision into individual feature influences for each sample. SHAP facilitates the visualization of feature importance within complex machine learning-based models, elucidating how individual features impact the likelihood of a particular output, either positively or negatively.

### Statistical analysis

Python3.7 was used for feature extraction, feature selection, machine learning model training, model evaluation, and plotting. SPSS (version 26.0) was used for the t-test, chi-square test, and Fisher’s exact test. The ROC curve was plotted for model performance evaluation. AUC was calculated, and bootstrapping was utilized for calculating the 95% confidence interval (95% CI). Continuous variables are presented as mean ± standard deviation, and categorical variables are presented as counts (%). Student’s t-test was used to compare continuous variables, and the chi-square test or Fisher’s exact test was used to compare categorical variables. Statistical significance was set at *p*<0.05.

## Results

### Patient population characteristics

The baseline clinical characteristics and pCR distribution of the patients in each cohort are summarized in **Tables 1** and **2**, respectively. Patients achieving pCR accounted for 28% (60/214) and 28.1% (25/89) in the radiomics and pathomics groups, respectively. There were no statistically significant differences in baseline characteristics between patients with and without pCR, except for sex (p=0.015) in the pathomics cohort.

**Table 1:** Clinical characteristics of patients.

Characteristics	Radiomics cohort (n=214)			Pathomics cohort (n=89)		
	pCR(n=60)	non-pCR (n=154)	P value	pCR(n=25)	non-pCR(n=64)	P value
**Age, mean, y**	62.6 ± 7.1	62.7 ± 6.5	0.933	64.6 ± 5.8	62.8 ± 7.3	0.291
Gender			0.153			0.015
Male	48 (80.0)	135 (87.7)		18 (72.0)	60 (93.8)	
Female	12 (20.0)	19 (12.3)		7 (28.0)	4 (6.2)	
Smoking status			0.302			0.257
Never	26 (43.3)	55 (35.7)		11 (44.0)	20 (31.3)	
Former or Current	34 (56.7)	99 (64.3)		14 (56.0)	44 (68.7)	
Histology			0.251			0.354
Adenocarcinoma	2 (3.3)	14 (9.1)		0 (0.0)	5 (7.8)	
Squamous cell carcinoma	58 (96.7)	140 (90.9)		25 (100.0)	59 (92.2)	
T stage			0.252			0.284
T1	1 (1.7)	2 (1.3)		0 (0.0)	1 (1.6)	
T2	7 (11.7)	8 (5.2)		5 (20.0)	6 (9.4)	
T3	50 (83.3)	133 (86.4)		20 (80.0)	52 (81.2)	
T4a	2 (3.3)	11 (7.1)		0 (0.0)	5 (7.8)	
N stage			0.386			0.091
N0	8 (13.3)	35 (22.7)		2 (8.0)	18 (28.1)	
N1	39 (65.0)	83 (53.9)		16 (64.0)	35 (54.7)	
N2	11 (18.4)	30 (19.5)		7 (28.0)	9 (14.1)	
N3	2 (3.3)	6 (3.9)		0 (0.0)	2 (3.1)	
TNM stage			0.621			0.06
I	1 (1.7)	2 (1.3)		0 (0.0)	1 (1.6)	
II	12 (20.0)	39 (25.3)		5 (20.0)	21 (32.8)	
III	43 (71.6)	97 (63.0)		20 (80.0)	34 (53.1)	
IVA	4 (6.7)	16 (10.4)		0 (0.0)	8 (12.5)	
Treatment cycles			0.961			0.541
≤2	45 (75.0)	116 (75.3)		22 (88.0)	51 (79.7)	
>2	15 (25.0)	38 (24.7)		3 (12.0)	13 (20.3)	
Tumor location			0.76			0.626
Upper segment	3 (5.0)	6 (3.9)		2 (8.0)	2 (3.1)	
Middle segment	24 (40.0)	55 (35.7)		8 (32.0)	23 (35.9)	
Lower segment	33 (55.0)	93 (60.4)		15 (60.0)	39 (60.9)	
PD-L1, CPS			0.238			0.396
<1	3 (5.0)	20 (13.0)		1 (4.0)	8 (12.5)	
≥1	14 (23.3)	33 (21.4)		7 (28.0)	19 (29.7)	
NE	43 (71.7)	101 (65.6)		17 (68.0)	37 (57.8)	

**Table 2:** pCR rate among different cohort.

		Radiomics cohort (n=214)	Pathomics cohort (n=89)	Radiopathomics cohort (n=80)
Training set	pCR	45	19	17
	non-pCR	115	48	43
	pCR rate	28.1%	28.4%	28.3%
Testing set	pCR	15	6	6
	non-pCR	39	16	14
	pCR rate	27.8%	27.3%	30%

### Feature selection and interpretation

**Figure 4:**
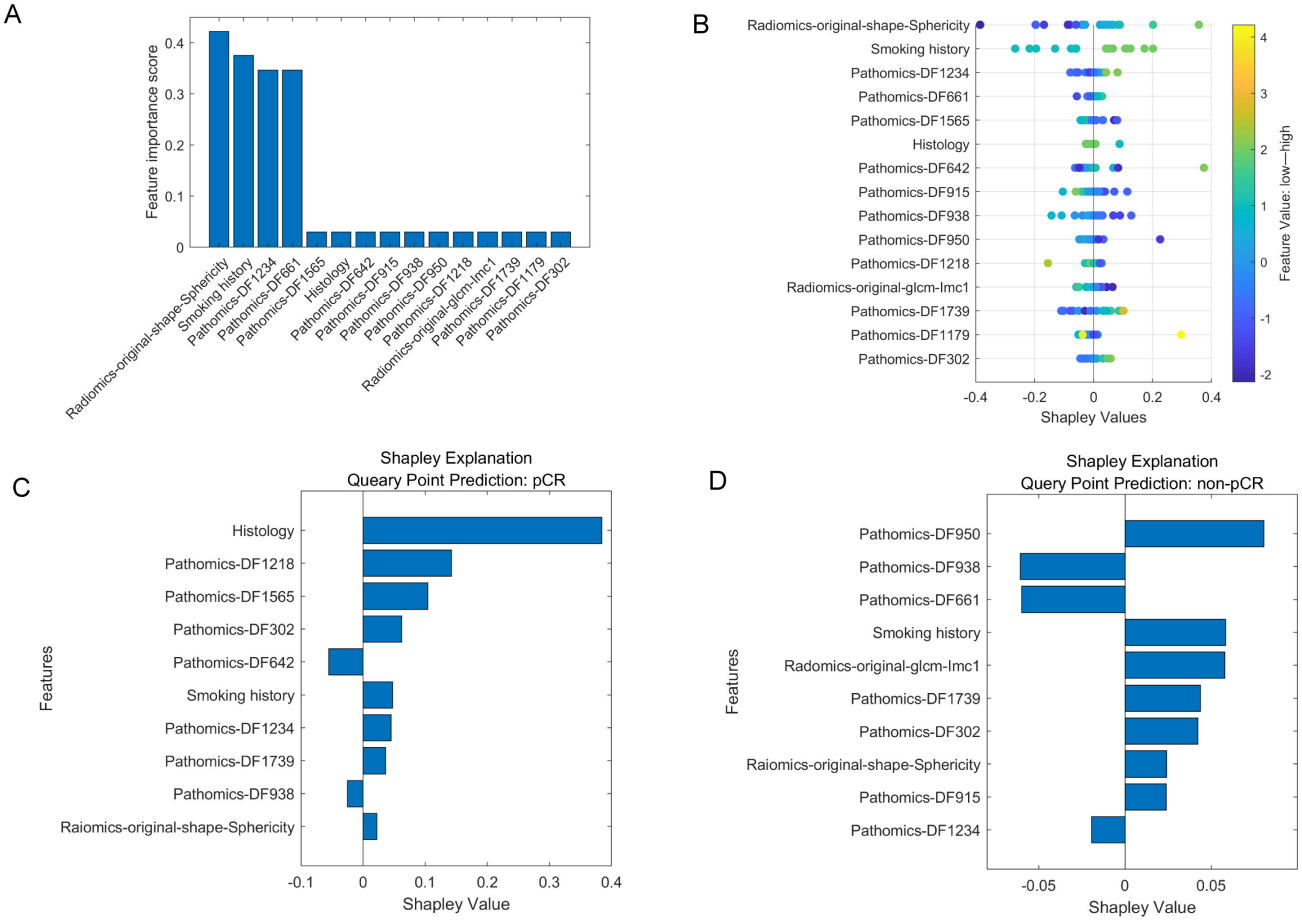
The selected features in multi-modality model. **(A)**. The importance scores of the top 15 features chosen by the MRMR. **(B)**. SHAP values of the multi-modality model for overall patient cohort. **(C, D)**. SHAP values of the multi-modality model for cases with pCR and non-pCR, respectively. A SHAP value larger than zero indicates a positive impact, while a SHAP value smaller than zero indicates a negative impact. The features with absolute SHAP values smaller than 0.01 were not shown in this figure.

In our study, 851 radiomics features were extracted from ROIs on CT images, and 2048 deep learning features (pathomics features) were extracted from pathological section images. We performed an independent feature-selection strategy for each feature set within the training cohort. Following dimensionality reduction, 10 radiomics features, 20 pathomics features, and 15 combined features significantly associated with pCR were retained. The selected features of these model are listed in **Table 3** and **Figure 4A**.

**Table 3:** The top 10 radiomics, 20 pathomics and 15 combined feature in the final model.

Radiomics feature	Pathomics feature	Combined features
wavelet-HHH_glszm_SmallAreaLowGrayLevelEmphasis	DF1122	original_shape_Sphericity
original_shape_Sphericity	DF97	original_glcm_lmc1
wavelet-LLH_glcm_Correlation	DF236	DF1234
wvelet-LHL_firstorder_Skewness	DF251	DF661
wavelet-LHL_glcm_Correlation	DF318	DF1565
wavelet-HLL_gldm_LargeDependenceHighGrayLevelEmphasis	DF849	DF642
wavelet-HHH_glszm_LowGrayLevelZoneEmphasis	DF989	DF915
wavelet-HHL_firstorder_Median	DF1331	DF938
wavelet-LHH_firstorder_Skewness	DF1679	DF950
wavelet-LHH_gldm_LargeDependenceLowGrayLevelEmphasis	DF1739	DF1218
	DF1892	DF1739
	DF938	DF1179
	DF302	DF302
	DF1300	Smoking history
	DF927	Histology
	DF1159	
	DF1731	
	DF941	
	DF1400	
	DF1565	

To further evaluate the contribution of the combined features to the multi-modality model prediction, the SHAP value was calculated to decompose the SVM model decision into individual feature influences for each sample. **Figure 4B** shows the SHAP values of the multi-modality model for overall patient cohort, as well as **Figure 4C, D** show the impact of the important features on the model output in one pCR case and one non-pCR case, respectively. For the pCR case, the “Histology” was the feature that contributed the most to the model output, while four pathomics features also showed great impact. For the non-pCR case, three pathomics features corresponded to an increased likelihood of non-pCR.

### Performance comparison of the prediction models

**Figure 5:**
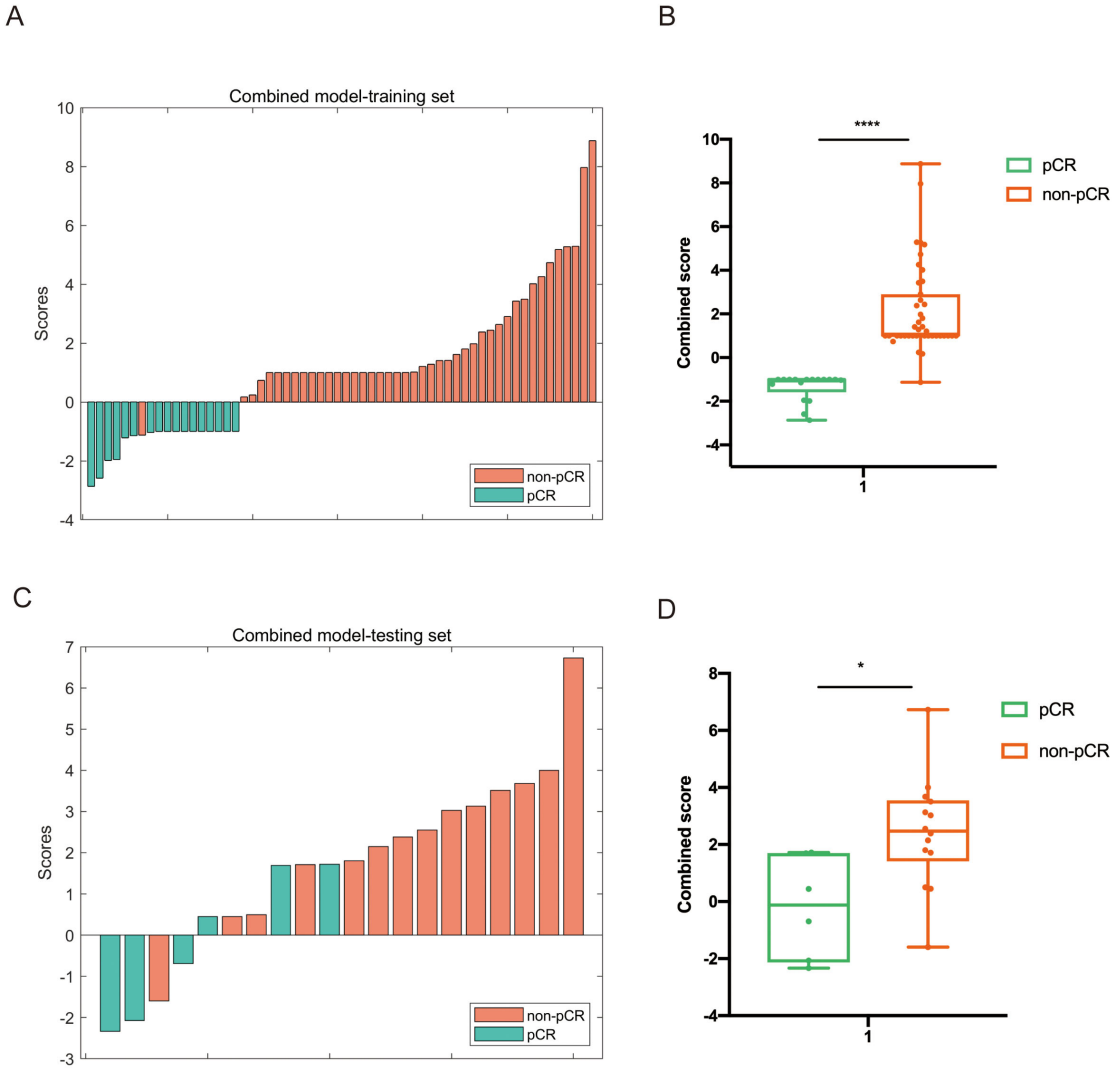
Distribution of the combined score between the pCR and non-pCR groups in the multi-modality model in both training **(A, B)** and testing set **(C, D)**.

By integrating clinical, radiomics, and pathomics features in the training set, a combined multi-modality prediction model was developed using the nonlinear SVM method. The distributions of combined scores are illustrated in **Figure 5**, with an increase in combined score, and more participants achieving pCR were identified in the training and testing cohorts.

**Figure 6:**
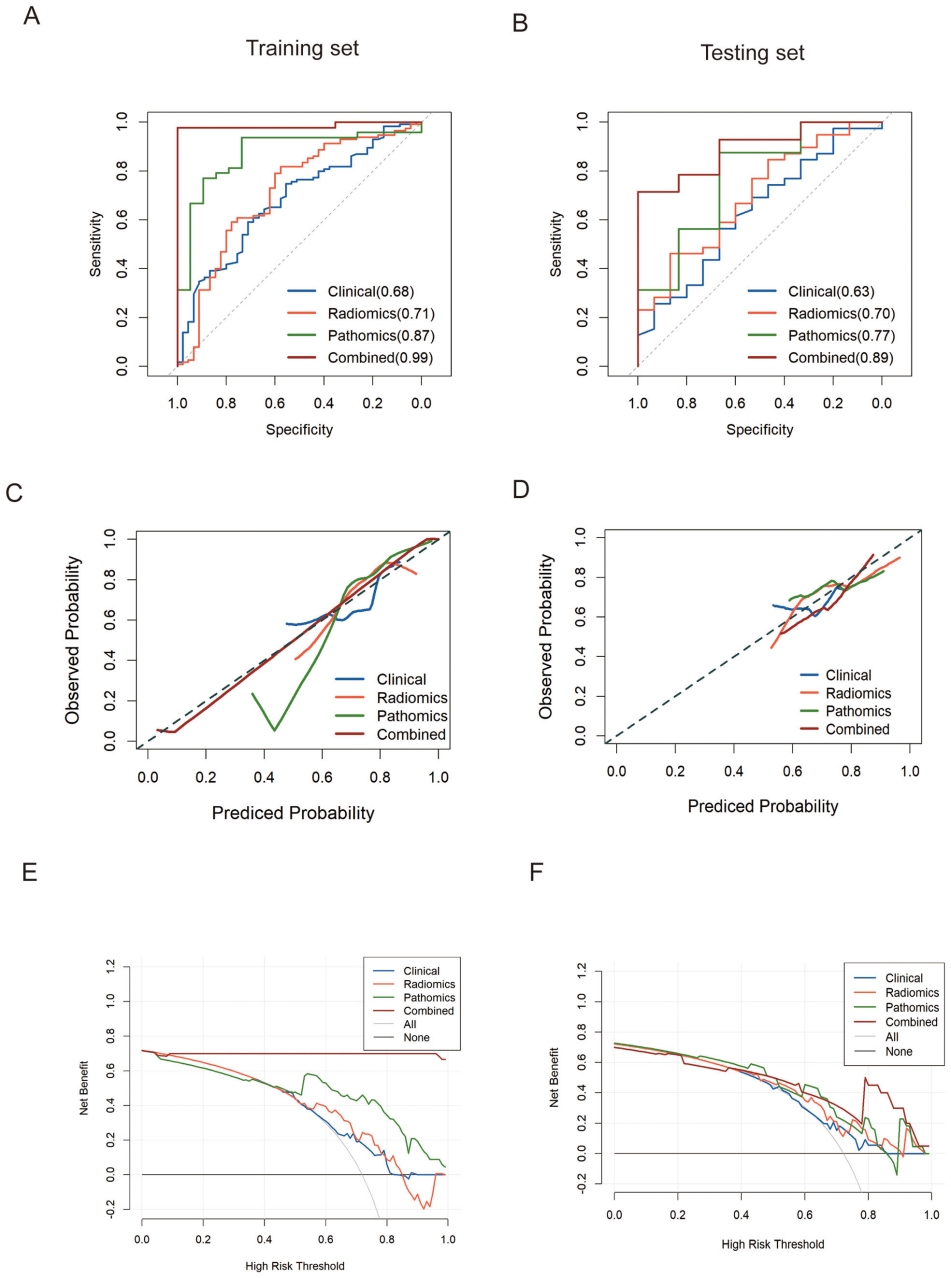
ROC analysis of predict models for predicting pCR in the training set **(A)** and validation set **(B)**, respectively. **(C, D)**. Calibration curves of models in training and testing set on discriminating pCR versus non-pCR. **(E, F)**. Decision curve analysis (DCA) was performed on four models for classifying pCR versus non-pCR in the training and testing cohorts.

**Table 4** and **Figure 6** present a comparison of the different SVM models. The combined model accurately predicted pCR in the training (AUC=0.99 [0.96-1.00]) and testing (AUC=0.89 [0.75-1.00]) sets. The specificity of the multi-modality was markedly high in the testing set at 94.28%, while the sensitivity was moderate (66.7%). The NPV of the combined model was 86.7% in the testing set, whereas the PPV was approximately 80%. Additionally, the single radiomics model displayed a marginally lower AUC (0.70 [0.54-0.85]) than multi-modality model, whereas the clinical model had a much lower AUC of 0.63 (0.46-0.80). The pathomics model yielded a higher AUC (0.77 [0.53-1.0]) than the other single-modality models but was still slightly lower than that of the multi-modality model (**Figures 6A, B**).

**Table 4:** The performance of the proposed models for the prediction of pCR.

Model	Training set						Testing set					
	AUC (95%CI)	ACC	SEN	SPE	PPV	NPV	AUC (95%CI)	ACC	SEN	SPE	PPV	NPV
Clinical model	0.68 (0.59-0.77)	69.4%	55.6%	74.8%	46.3%	81.1%	0.63 (0.46-0.80)	66.7%	46.7%	74.4%	41.2%	78.4%
Radiomics model	0.71 (0.62-0.81)	75.0%	57.8%	81.7%	55.3%	83.2%	0.70 (0.54-0.85)	72.2%	40.0%	84.6%	50%	78.6%
Pathomics model	0.87 (0.78-0.97)	88.1%	73.7%	93.8%	82.4%	90.0%	0.77 (0.53-1.00)	77.3%	66.7%	81.3%	57.1%	86.7%
Combined model	0.99 (0.96-1.00)	95%	100.0%	93.0%	85%	100%	0.89 (0.75-1.00)	85%	66.7%	92.9%	80%	86.7%

AUC, Area under the receiver operating curve; CI, Confidence interval; SEN, sensitivity; SPE, Specificity; ACC, Accuracy; PPV, Positive predictive value; NPV, Negative predictive value.

### Clinical usefulness of the multi-modality model

We evaluated the clinical usefulness of the combined multi-modality model for pCR recognition via DCA in the training and testing cohorts, revealing that the multi-modality model conferred better net benefits than the CT radiomics and pathomics models (**Figure 6E, F**). The calibration curves also revealed that the prediction probability of the multi-modality model was in good agreement with the actual outcomes of neoadjuvant chemoimmunotherapy for esophageal cancer in both the training and testing groups (**Figures 6C, D**).

## Discussion

We developed a combined predictive model using multimodal pretreatment CT radiomics and WSI pathomics features, in addition to clinical variables, to predict the response to neoadjuvant chemoimmunotherapy in esophageal cancer prior to treatment initiation. The integrative, multi-modality model showed significant improvement in assessing the response to neoadjuvant chemoimmunotherapy compared with the single-modality model in terms of the AUC (0.89 [95% CI: 0.75,1.00]). To our knowledge, this is the first study to combine pathomics and radiomics in the field of esophageal cancer.

Individualized treatment for patients with esophageal cancer undergoing neoadjuvant therapy has been a research priority over the past decade. Neoadjuvant chemoimmunotherapy has shown remarkable advances in the management of esophageal cancer, resulting in a high rate of pCR and improved long-term prognosis (8, 23, 24). However, pCR can only be confirmed postoperatively. Patients who are sensitive to neoadjuvant chemoimmunotherapy might have good prognoses and could benefit from watch-and-wait strategies (25). Conversely, for patients who do not respond to neoadjuvant chemoimmunotherapy, earlier surgical intervention can be considered to avoid unnecessary immunotherapy-associated toxicity and morbidity. Recently, several studies have explored the predictive effectiveness of genetic (26, 27), microbiome (28), and tumor microenvironment biomarkers (3, 29) in EC patients following neoadjuvant chemoimmunotherapy. However, their clinical application remains limited due to high costs and invasiveness. Therefore, the development of a preoperative and accurate approach to predict pCR prior to treatment is meaningful.

Radiomics is a rapidly developing research field, and many studies have demonstrated the role of radiomics biomarkers in predicting pCR to neoadjuvant chemoradiotherapy in esophageal cancer (11, 30, 31). A meta-analysis by Zhao et al. (32) highlighted the clinical value of pretreatment imaging-based radiomics in predicting pCR, achieving a pooled AUC of 0.84 (95%CI: 0.81–0.87) in patients who underwent neoadjuvant chemoradiotherapy. However, the role of radiomics in predicting pCR following neoadjuvant chemoimmunotherapy for esophageal cancer remains limited. In our study, the AUC value of the prediction model based on radiomics features was only 0.70 in the testing set, which was lower than that reported by Li et al. (33). The superior results in Li et al.’s study was mainly attributed to post-treatment CT, which provided direct information on tumor regression or residual after treatment. However, their model could not provide an earlier estimation of treatment response to guide the administration of neoadjuvant chemoimmunotherapy and required patients to undergo several examinations. Thus, the predictive performance of the single-modality model based solely on radiomics features remains unsatisfactory. Radiomics-derived data should be combined with other features to achieve a more powerful predictive value (34).

In contrast to radiomics, which captures the spatial macrostructure of tumors, histopathology contains valuable microstructural information about the tumor cell, extracellular matrix, and tissue morphology, which may be overlooked in radiomics analyses. With the development of artificial intelligence and digital pathology, we can extract quantitative features from diagnostic slides that cannot be recognized by the naked eye (35–38). Pathomics-based analyses of esophageal cancer specimens have proven effective in lesion detection, cancer diagnosis, and prognostic evaluation (14, 15). In terms of predicting the efficacy of neoadjuvant therapy, Bhargava (39) and Tian (40) et al. demonstrated that the pathological features from biopsy slides are predictive of pCR in breast cancer with an AUCs of 0.71-0.73 in the validation cohorts. Consistent with these studies, our pathomics model based on deep learning also demonstrated good performance in predicting pCR in esophageal cancer (AUC of 0.87 on the training set and 0.77 on the testing set). Biopsies can provide insights into specific subregions or areas with unique characteristics of the tumor tissue, such as cellular composition and morphological features, under a microscope. By fusing biopsy data with imaging, complementary information from both microscopic and macroscopic levels representative of tumor heterogeneity can be provided. The superior performance of our combined multi-modality model is likely due to the integration of heterogeneous radiomics and pathomics features, suggesting that the comprehensive capture of microstructural and macrostructural features can effectively predict pCR in esophageal cancer.

In clinical practice, the combined multi-modality model has the potential to assist clinicians in accurately predicting the efficacy of neoadjuvant chemoimmunotherapy before its administration, which is critical for developing patient treatment plans and optimizing overall patient management. For patients predicted to achieve a pCR, it may be beneficial to administer intensive neoadjuvant chemoimmunotherapy and adopt a watch-and-wait strategy to improve survival and quality of life. Conversely, for patients unlikely to achieve a pCR, neoadjuvant treatment might be unnecessary due to its associated excessive treatment-related toxicity.

Despite the innovative nature of our study, several limitations exist. First, the study’s retrospective design, along with the small sample size and absence of external validation. A total of 223 patients were included in our study; however, combined pathological and radiological analysis was only conducted in 80 patients due to the exclusion of a significant number of patients who did not undergo biopsy at our institution. Multicenter and large-sample prospective studies are warranted to further validate the preliminary results. Another significant factor that hampers the effectiveness of the proposed prediction model is the imbalance in the distribution of baseline characteristics, such as sex, between patients who achieved a pCR and those who did not within the pathomics cohort. Specifically, the relatively small number of females in our study may influence the outcomes, as previous literature has indicated that sex is associated with the extent of benefit derived from immunotherapy (41). Our future research will include more female patients to ensure the balance of baseline characteristics between patients with and without pCR. Additionally, previous studies (4) have shown that adenocarcinoma (ADC) and squamous cell carcinoma (SCC) have different response to neoadjuvant chemoimmunotherapy and may lead to different prediction performances. The model constructed by merging the two histopathological types may lead to a decline in prediction efficiency. The lack of adequate patients with ADC (less than 10%) also placed a higher weight on the SCC group, which may bias the pooled estimates toward SCC group when generating the overall results. Therefore, larger data sets should be used in future research, and the two histological subtypes should be hierarchically modeled and verified. Finally, all ROIs are manually delineated from the arterial-phase CT images, which is time-consuming and laborious. It is necessary to collect multiphase CT data and develop a user-friendly tool to encourage the use of radiomic measures in daily clinical practice.

In conclusion, the current study demonstrated that a model integrating pretreatment CT, WSI, and clinical variables significantly enhances the prediction of pCR in patients undergoing neoadjuvant chemoimmunotherapy, compared to single-modality models. The improved performance of our multi-modality model highlights its potential for advancing precision decision-making and personalizing treatment strategies for patients with esophageal cancer.

## Data Availability

The original contributions presented in the study are included in the article/supplementary material. Further inquiries can be directed to the corresponding authors.
